# Parental Knowledge, Awareness, and Attitude Regarding Children With Epilepsy: A Cross-Sectional Study in Arar, Saudi Arabia

**DOI:** 10.7759/cureus.73230

**Published:** 2024-11-07

**Authors:** Rouh Maskhur K Alanazi, Ayman Alenezi, Razan I Alsayer, Maram M Alenezi, Norah S Alenazi, Ahad Khalifah Alanazi

**Affiliations:** 1 Faculty of Medicine, Northern Border University, Arar, SAU; 2 Pediatric Medicine, Maternity and Children Hospital, Arar, SAU

**Keywords:** arar, attitudes, awareness, epilepsy, knowledge, neurology, parents, pediatric, saudi arabia, seizure

## Abstract

Background: Epilepsy is a chronic neurological condition marked by recurrent seizures, which differ widely in type and frequency. Globally, many families lack accurate information about epilepsy and may be misinformed by unreliable sources, often resulting in negative attitudes toward their children who have epilepsy. The aim of this study is to assess parental knowledge and attitude regarding epilepsy and identify the predictors affecting the knowledge and attitude in Arar city, Saudi Arabia.

Methods: A cross-sectional study was conducted in Arar, Saudi Arabia, among 66 parents. A self-administered online questionnaire was disseminated between January and April 2024, and responses were collected anonymously. Surveys were distributed at the Maternity and Children Hospital in Arar, Saudi Arabia.

Results: The Cronbach's alpha for the knowledge domain was 0.55, indicating moderate reliability, and for the attitude domain it was 0.42, indicating poor reliability. Principal component analysis revealed that the knowledge domain loaded on two components explaining 59.44% of the variance in responses and the attitude domain loaded on three components explaining 61.16% of the variance in responses. Of the total respondents, 11 (16.7%) had good knowledge, 13 (19.7%) had fair knowledge, and 42 (63.6%) had poor knowledge. Of the total respondents, 38 (57.5%) had a good attitude, 17 (25.8%) had a fair attitude, and 11 (16.7%) had a poor attitude. The multiple linear regression analysis for the knowledge score revealed that a child's treatment duration of 6-10 years significantly increased the knowledge score by 1.75 (95%CI: 0.28 - 3.23, p=0.021). The attitude score revealed that a secondary or higher educational level significantly improved it by 1.95 (95%CI: 0.03 - 3.86, p=0.046), while an intermediate educational level non-significantly improved the attitude score by 1.40 (95%CI: -0.99 - 3.78, p=0.245). Employment status significantly decreased the attitude score by -0.79 (95%CI: -1.34 - -0.24, p=0.006). Additionally, a treatment duration of 6-10 years significantly improved the attitude score by 1.21 (95%CI: 0.12 - 2.29, p=0.030).

Conclusion: Our study highlights a notable disparity between poor knowledge and positive attitudes among the parents surveyed. This underscores the imperative for targeted health education initiatives and awareness campaigns aimed at equipping parents with a comprehensive understanding of their children's condition.

## Introduction

Epilepsy is a complex neurological disorder defined by the occurrence of recurrent, unprovoked seizures that differ in type, intensity, and frequency. Seizures are triggered by irregular electrical activity in the brain, which can interfere with normal brain function and result in a range of physical and behavioral symptoms [1]. Childhood epilepsy often requires not only careful medical management but also specialized educational and psychosocial support to address its far-reaching effects [2]. In 2013, The European Forum on Epilepsy Research recommended reducing the social burden associated with epilepsy by increasing awareness and decreasing the treatment gaps [2,3].

Many children with epilepsy around the world do not attend school due to widespread misconceptions, a lack of awareness, and negative attitudes regarding the condition [4]. Epilepsy is sometimes misunderstood as incurable or contagious, causing these children to feel alone, ashamed, and embarrassed. This social stigma can result in psychiatric comorbidities like depression and anxiety [4]. When a child is diagnosed with epilepsy, it can cause confusion and anxiety among parents, especially if they have limited knowledge about the condition. It is crucial for parents to have accurate information about epilepsy and its implications for their child's overall well-being [5]. Understanding the knowledge, awareness, and attitudes of parents regarding children with epilepsy is essential for providing the appropriate support and care these young individuals need [6].

In recent decades, there has been increased global awareness of epilepsy's impact on children. Many studies assess the general knowledge and attitude about epilepsy, although parental knowledge and attitude have the most impact on the lives of children with epilepsy [7]. Parents' attitudes may impact children's epilepsy complications. Providing information and raising awareness among parents can help children with epilepsy manage their symptoms [8]. Worldwide, many studies reported poor knowledge with many misconceptions, more commonly in developing countries [9,10]. Mohamed et al.'s study in Egypt reported poor knowledge in 71.9% of participants and 85% of them had a negative attitude towards epilepsy [11]. In the study by Zainy et al. conducted in the King Abdulaziz University Hospital, poor knowledge and negative attitudes of parents were seen regarding their children with epilepsy. They focused on many misconceptions such as epilepsy being a mental disease, or that it is evil [12]. Most Saudi parents have poor knowledge regarding epilepsy which results in poor attitudes and many conceptions need to be corrected [13,14].

Although many studies have been conducted in Saudi Arabia, there is still room for improvement in public awareness and perceptions in Saudi Arabia, and research on parental understanding is scarce [8]. Many families in Saudi Arabia lack proper information about epilepsy and are sometimes misinformed by unreliable sources, leading to negative attitudes towards their children with epilepsy [12,15]. The aim of this study was to assess parental knowledge and attitudes regarding epilepsy in Arar city, Saudi Arabia, and identify the predictors affecting their knowledge and attitudes.

## Materials and methods

This was a cross-sectional study conducted at the Maternity and Children Hospital in Arar, Saudi Arabia, and responses were collected anonymously. The study was approved by the Local Committee of Bioethics of Northern Border University (approval number: 6/24/H). Informed consent was obtained from all participants before completing the questionnaire. Participants were assured of the confidentiality of their responses.

Study population

The study included parents of both genders, aged 18-60 years, residing in Arar, Saudi Arabia, who had at least one child diagnosed with epilepsy, and expressed voluntary willingness to engage in the research. Parents who did not have children with epilepsy, were not from Arar city, or were unwilling to provide informed consent were excluded. This was achieved by having specific preliminary questions to confirm their eligibility, which were given to all parents. These questions included whether they were residents of Arar, if they had a child diagnosed with epilepsy, and if they were willing to provide informed consent. Respondents who did not meet these criteria were automatically redirected away from the main questionnaire and thanked for their willingness to participate.

Sample size calculation and sampling

The minimal sample size was calculated to be 63 according to the single proportion sample size formula to achieve 80% power at a 95% significance level, considering the expected prevalence of epilepsy in Saudi Arabia as 0.00654 [16]. A total of 66 responses were collected through the non-probability cluster-convenient sampling technique.

Data collection tool

The survey instrument utilized was a modified version of the questionnaire employed in a previous study conducted by Alharthi et al. in the Al Baha Region of Saudi Arabia, for assessing parental knowledge and attitudes towards epilepsy in Saudi Arabia [9], and was specifically tailored for use in the Arar region. This tool underwent adjustments to align with the study's focus on cultural context and local nuances. While the knowledge domain yielded a Cronbach's alpha of 0.55 (moderate reliability) and the attitude domain a lower alpha of 0.42 (poor reliability), principal component analysis (PCA) demonstrated strong construct validity, with the knowledge domain explaining 59.44% of the response variance and the attitude domain 61.16%. The knowledge domain assessed parental understanding of epilepsy. The attitude domain, meanwhile, explored parental beliefs regarding the nature of epilepsy.

The self-administered online questionnaire was disseminated to 74 parents who fulfilled the inclusion and exclusion criteria, between January and April 2024 at the Maternity and Children Hospital, and responses were collected anonymously.

Statistical analysis

IBM SPSS Statistics for Windows, Version 24.0 (Released 2016; IBM Corp., Armonk, New York, United States) was used for descriptive analysis of categorical data as percentages. Cronbach alpha was used to assess the reliability of the questionnaire, and principal component analysis was used to identify underlying patterns or structures within the data. Domain scores were calculated considering 70-100% as a good level of knowledge or attitude, 69-50% as a fail level, and less than 50% as a poor level. Multiple linear regression models were used to identify the predictors of knowledge and attitude, after testing the assumptions of linearity and multicollinearity, choosing the best-fitted model based on the lowest Akaike Information Criterion (AIC). Statistical significance was defined as p-values of less than 0.05.

## Results

Of the 74 surveys distributed to parents/guardians having at least one child with an epilepsy diagnosis, 66 were completed and included in the final analysis. Of these, 47 (71.2%) were completed by mothers, while only four (6.1%) were completed by fathers. The majority of respondents (n=25, 37.9%) were aged 31-40 years. Additionally, 63 (95.5%) participants had attained at least a secondary level of education, and 45 (68.2%) were employed (Table 1).

**Table 1:** Family characteristics of participants (N=66)

Family characteristics	Categories	Frequency (Percentage)
Respondent	Aunt	3 (4.5)
	Father	4 (6.1)
	Mother	47 (71.2)
	Sister	5 (7.6)
	Uncle	3 (4.5)
	Missing	4 (6.1)
Age group (years)	18-30	24 (36.4)
	31-40	25 (37.9)
	41-50	11 (16.7)
	51-60	6 (9.1)
Education level	Primary	1 (1.5)
	Intermediate	2 (3)
	Secondary or above	63 (95.5)
Work status	Not working	21 (31.8)
	Working	45 (68.2)

Most of the respondents (n=32, 48.5%) had children aged one to five years. Additionally, 26 (39.4%) children had been diagnosed with epilepsy within the past one to five years. Generalized seizures were reported in 29 (43.9%) children, while five (7.6%) had experienced treatment involving beating or cautery. Levetiracetam (Keppra™) was being used to treat 27 (40.9%) children. Notably, 53 (80.3%) children did not have any other family members diagnosed with epilepsy (Table 2). 

**Table 2:** Characteristics of the children with epilepsy of the participants (N=66)

Child characteristics	Category	Frequency (Percentage)
Child's age	1-5 years	32 (48.5)
	6-10 years	16 (24.2)
	11-15 years	9 (13.6)
	16-18 years	9 (13.6)
Duration of since diagnosis	Less than one year	23 (34.8)
	1-5 years	26 (39.4)
	6-10 years	10 (15.2)
	More than 10 years	7 (10.6)
Duration of the treatment	Less than one year	27 (40.9)
	1-5 years	28 (42.4)
	6-10 years	6 (9.1)
	More than 10 years	5 (7.6)
Epilepsy type	Partial seizure	25 (37.9)
	Generalized seizure	29 (43.9)
	Others	12 (18.2)
Did you ever treat your child by beating or cautery?	Yes	5 (7.6)
	No	61 (92.4)
Treatment type	Keppra™ (levetiracetam)	27 (40.9)
	Depakine™ (valporate sodium)	16 (24.2)
	Topamex™ (topiramate)	16 (24.2)
	Others	7 (10.6)
Is there any other family member diagnosed with epilepsy?	Yes	13 (19.7)
	No	53 (80.3)

PCA revealed that the knowledge domain loaded on two components: the first component explained 37.17% of the variance in the responses, and the second component explained 22.27% (Table 3). In the attitude domain, the first component explains 24.86% of the variance, followed by the second component with 18.49%, and the third with 17.80%, accounting for a total cumulative variance of 61.16% (Table 4).

**Table 3:** PCA for Knowledge domain PCA: principal component analysis

PCA for knowledge domain	Eigen value	Percentage of explained variance	Accumulated percentage of explained variance
Component 1	2.230	37.171	37.171
Component 2	1.337	22.278	59.449

**Table 4:** PCA for Attitude domain PCA: principal component analysis

PCA for attitude domain	Eigen value	Percentage of explained	PCA for attitude domain
Component 1	1.740	24.862	24.862
Component 2	1.295	18.495	43.358
Component 3	1.246	17.805	61.163

**Figure 1:**
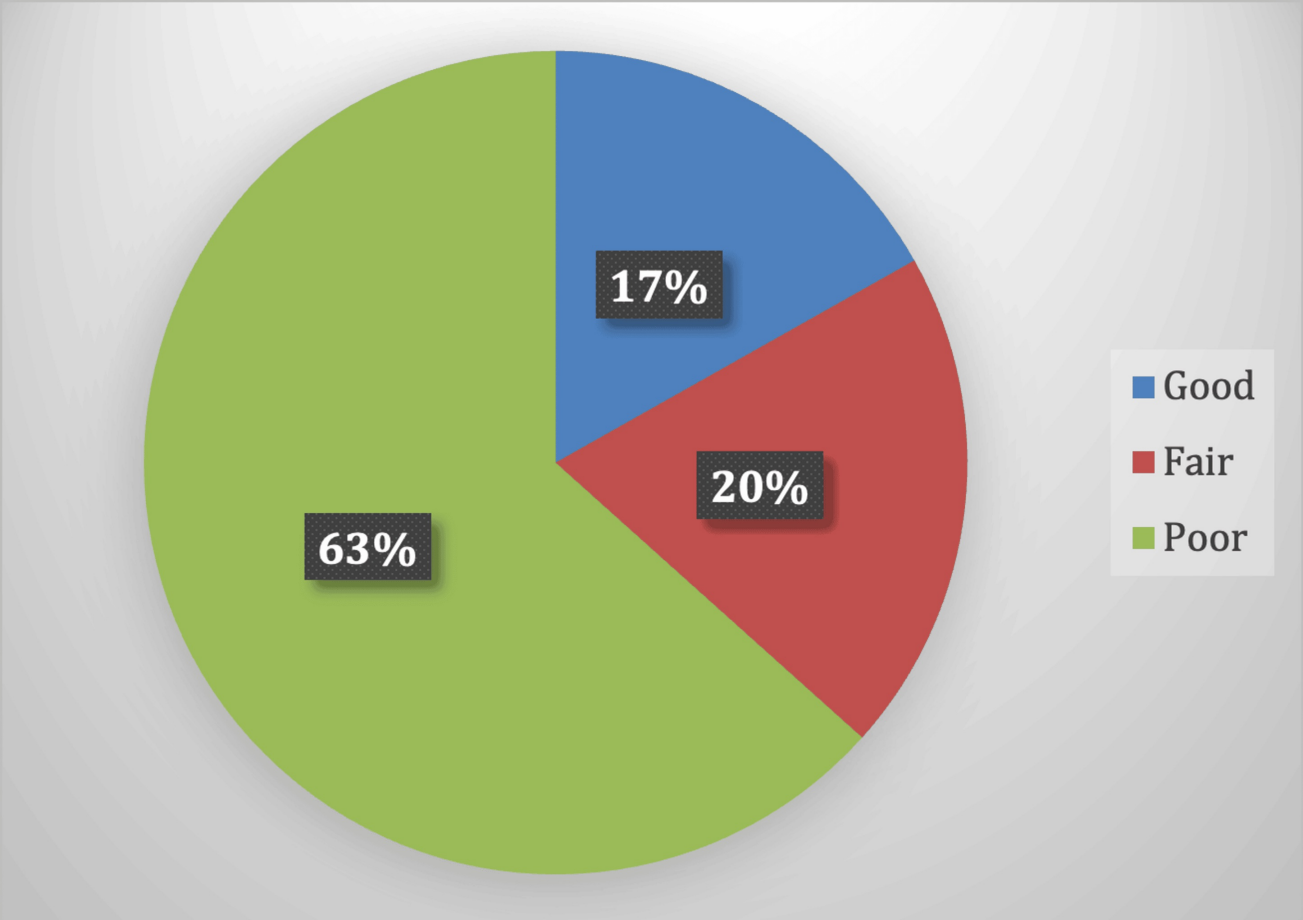
Distribution of Knowledge scores

Exactly 65 (87.8%) respondents believed that children with epilepsy require special treatment, suggesting that most respondents either did not perceive a need for specialized care or were unaware of such options. Regarding sports participation, 51 (68.9%) respondents felt that children with epilepsy could engage in any type of sport. The impact of epilepsy medication on various aspects of children's lives was also a significant concern among respondents. Nearly half (n=35, 47.3%) reported that epilepsy drugs negatively affected children's school achievement, while a larger proportion of respondents (n=47, 63.5%) believed that these medications impacted the child’s overall activity levels. Additionally, 42 (56.8%) confirmed that epilepsy drugs have a broader negative effect on the patient’s life, highlighting concerns about side effects and their implications for life. Before removing the third question, the knowledge scores were distributed as follows: 25 (37.9%) participants were classified as having poor knowledge, 18(27.3%) as fair, and 23(34.8%) as good (Table 5). However, after excluding the third question, the distribution shifted significantly, with 42 (63.6%) participants categorized as having poor knowledge, 13 (19.7%) as fair, and only 11 (16.7%) as good (Figure 1).

**Table 5:** Scores in the Knowledge domain

	Knowledge questions	Responses	Frequency (Percentage)
Q1	Do you think non-medical treatment (beating and cautery) should be considered for epilepsy?	Yes	11 (14.9)
		No (correct)	63 (85.1)
Q2	Do you think that children with epilepsy need special treatment?	Yes	65 (87.8)
		No (correct)	9 (12.2)
Q3	Do you think an epileptic child has the capability for school achievement?	Yes (correct)	70 (94.6)
		No	4 (5.4)
Q4	Do you think that a child with epilepsy can participate in any type of sport?	Yes	51 (68.9)
		No (correct)	23 (31.1)
Q5	Do you think epileptic drugs affect patient school achievement?	Yes (correct)	35 (47.3)
		No	39 (52.7)
Q6	Do you think that epilepsy drugs affect the child's activity?	Yes (correct)	47 (63.5)
		No	27 (36.5)
Q7	Do you think epileptic drugs affect patient life?	Yes (correct)	42 (56.8)
		No	32 (43.2)
Knowledge score with Q3		Poor	25 (37.9)
		Fair	18 (27.3)
		Good	23 (34.8)
Knowledge score without Q3		Poor	42 (63.6)
		Fair	13 (19.7)
		Good	11 (16.7)

**Figure 2:**
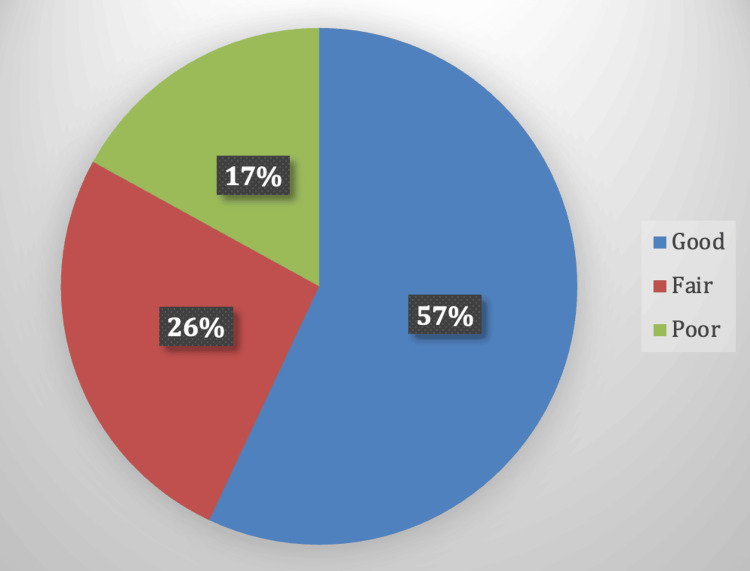
Distribution of Attitude scores

Considering specific attitude questions, 44 (59.5%) respondents didn’t know the cause of epilepsy, 67 (90.5%) believed that epilepsy is not contagious, 41 (55.4%) thought it is a heritable disease, 48 (64.9%) believed it is not a mental illness, 65 (87.8%) thought it is a curable disease, 61 (82.4%) knew the correct first aid for a child having an epileptic seizure, and 68 (91.9%) considered that drugs are the best treatment for epilepsy in children (Table 6). The distribution of attitude levels among respondents was as follows: 38 (57.5%) had good attitudes, 17 (25.8%) had fair attitudes, and 11 (16.7%) had poor attitudes (Figure 2). 

**Table 6:** Scores of the Attitude domain

	Attitude questions	Responses	Frequency (Percentage)
Q1	Do you know what causes epilepsy?	Yes	30 (40.5)
		No (correct)	44 (59.5)
Q2	Do you think epilepsy is a contagious disease?	Yes	7 (9.5)
		No (correct)	67 (90.5)
Q3	Do you think epilepsy is a hereditary disease?	Yes (correct)	41 (55.4)
		No	33 (44.6)
Q4	Do you think epilepsy is a mental illness?	Yes	26 (35.1)
		No (correct)	48 (64.9)
Q5	Do you believe that epilepsy is a curable disease?	Yes	65 (87.8)
		No (correct)	9 (12.2)
Q6	Do you know what is the correct first aid for a child having an epileptic seizure?	Yes (correct)	61 (82.4)
		No	13 (17.6)
Q7	What is the best treatment?	Drugs (correct)	68 (91.9)
		Surgery	3 (4.1)
		Others	3 (4.1)
	Attitude score	Poor	11 (16.7)
		Fair	17 (25.8)
		Good	38 (57.5)

The multiple linear regression analysis for the knowledge score revealed that a child's treatment duration of 6-10 years significantly increased the parental knowledge score by 1.75 (95%CI: 0.28 - 3.23, p=0.021). In contrast, treatment durations of one to five years and more than 10 years increased knowledge scores non-significantly by 0.42 (95%CI: -0.42 - 1.26, p=0.321) and 0.84 (95%CI: -0.60 - 2.29, p=0.248), respectively. Additionally, the knowledge score increased non-significantly with intermediate education by 0.65 (95%CI: -2.99 - 4.29, p=0.723) and with secondary or above education by 0.77 (95%CI: -2.14 - 3.68, p=0.599). The respondent's age non-significantly decreased the knowledge score for age groups older than 18-30 years (Table 7). 

**Table 7:** Multiple linear regression for the predictors of knowledge score

Predictors for Knowledge Score	Categories	Estimate (95%CI)	p-value
Intercept		2.00 (-0.83 – 4.83)	0.163
Respondent age group	18 – 30 years	Reference	
	31 – 40 years	-0.07 (-0.95 – 0.81)	0.874
	41 – 50 years	-0.40 (-1.46 – 0.66)	0.453
	51 – 60 years	-1.02 (-2.49 – 0.44)	0.168
Education level	Primary	Reference	
	Intermediate	0.65 (-2.99 – 4.29)	0.723
	Secondary or above	0.77 (-2.14 – 3.68)	0.599
Treatment duration	Less than one year	Reference	
	1 – 5 years	0.42 (-0.42 – 1.26)	0.321
	6 – 10 years	1.75 (0.28 – 3.23)	0.021*
	More than 10 years	0.84 (-0.60 – 2.29)	0.248

The multiple linear regression analysis for the attitude score revealed that neither respondent's age nor the child's age significantly affected the attitude. However, having a secondary or higher educational level significantly improved the attitude score by 1.95 (95%CI: 0.03 - 3.86, p=0.046), while an intermediate educational level non-significantly improved the attitude score by 1.40 (95%CI: -0.99 - 3.78, p=0.245). Employment status significantly decreased the attitude score by -0.79 (95%CI: -1.34 - -0.24, p=0.006). Additionally, a treatment duration of 6-10 years significantly improved the attitude score by 1.21 (95%CI: 0.12 - 2.29, p=0.030). Conversely, treatment durations of one to five years and more than 10 years did not significantly improve the attitude score, with values of 0.22 (95%CI: -0.38 - 0.82, p=0.468) and 0.89 (95%CI: -0.32 - 2.11, p=0.147), respectively (Table 8). 

**Table 8:** Multiple linear regression for the predictors of attitude score

Predictors for Attitude Score	Categories	Estimate (95%CI)	p-value
Intercept	-	3.00 (1.17 – 4.83)	0.002*
Respondent age group	18- 30 years	Reference	
	31-40 years	0.23 (-0.38 – 0.84)	0.547
	41-50 years	-0.00 (-0.78 – 0.78)	0.995
	51-60 years	-0.53 (-1.53 – 0.47)	0.295
Education level	Primary	Reference	
	Intermediate	1.40 (-0.99 – 3.78)	0.245
	Secondary or above	1.95 (0.03 – 3.86)	0.046*
Working status	Not working	Reference	
	Working	-0.79 (-1.34 – -0.24)	0.006*
Child age group	1-5 years	Reference	
	6-10 years	-0.05 (-0.67 – 0.57)	0.868
	11-15 years	-0.35 (-1.17 – 0.47)	0.393
	16-18 years	-0.15 (-1.16 – 0.86)	0.768
Treatment duration	Less than 1 year	Reference	
	1-5 years	0.22 (-0.38 – 0.82)	0.468
	6-10 years	1.21 (0.12 – 2.29)	0.030*
	More than 10 years	0.89 (-0.32 – 2.11)	0.147

## Discussion

Knowledge and attitudes regarding epilepsy in children play a fundamental role in fostering a balanced society. This study aimed to assess the knowledge, attitudes, and predictors of epilepsy among parents in Arar, Saudi Arabia. Our findings suggest that while the reliability of the knowledge scale was moderate and the reliability of the attitude domain was poor, the scale effectively captures the construct it is intended to measure in terms of validity. The discrepancy between reliability and validity could be due to that questionnaire items covered a broad range of topics related to epilepsy, leading to variability in responses, or the cultural factors and varied personal experiences with epilepsy among respondents might have contributed to diverse interpretations and answers to the questions. Despite the moderate and poor reliability for knowledge and attitude, respectively, the high validity indicated by PCA suggests that each domain captures essential aspects of parental knowledge and attitude about epilepsy.

The study findings revealed that 63.6% of family members had poor knowledge, while 57.5% exhibited a good attitude towards children with epilepsy. Poor knowledge of epilepsy has been observed in many developing countries [17,18]. In our study, 87.8% of respondents believed that children with epilepsy required specialized treatment, and 14.9% supported non-medical treatments such as beating and cautery. In contrast, Alharthi et al., in Al Baha Region, Saudi Arabia, reported that only 51% of families with children with epilepsy believed in the need for specialized treatment, and a lower percentage, 8.2%, endorsed non-medical treatments [9]. Regarding attitudes towards epilepsy, our findings indicate that 9.5% of respondents believed epilepsy to be contagious, and 35.1% considered it a mental illness. In contrast, Alharthi et al. found that none of the respondents with children who had epilepsy believed it to be contagious, although 75% thought it was a mental illness [9]. Our findings align with that of Alharthi et al. regarding the beliefs about the curability and hereditary nature of epilepsy, with reported rates of 81.6% and 55.1%, respectively [9]. However, Zainy et al. reported that 48% of respondents in their study considered epilepsy a mental illness and 44% associated it with evil [12]. This differs from our findings but aligns with our results concerning the curability and contagious nature of epilepsy, with 9% and 2% of their respondents considering it curable or contagious, respectively. 

Our findings suggest that a good attitude is associated with 6-10 years of treatment (1.21, 95%CI: 0.12 - 2.29, p=0.030) and a secondary or higher education level (1.95, 95 CI: 0.03 - 3.86, p=0.046). The importance of education level as a predictor of attitude has been demonstrated in previous studies [19]. Elsakka et al. conducted a study in Egypt using a different questionnaire, which revealed that 83.3% of participants had poor knowledge about epilepsy, 62.7% exhibited a negative attitude towards the condition, and 74.3% demonstrated poor practice skills in managing seizures [4]. These findings align with our results regarding poor knowledge but differ from our findings in terms of the reported attitudes, where our study did not observe the same level of negativity. On the contrary, Hassan et al.'s study in Abha City, Saudi Arabia, reported adequate awareness and attitude about epilepsy among parents, which may be due to different tools used to assess knowledge and attitude [8].

Although many studies link poor knowledge to poor attitudes, our findings do not support this concept. This discrepancy could be attributed to cultural influences, where attitudes towards individuals with medical conditions are more strongly shaped by cultural norms and societal values than by specific knowledge about the disease. Additionally, positive attitudes may arise from personal or familial experiences with epilepsy, even if formal knowledge is limited. In conclusion, knowledge and attitude are not inherently connected to each other [20].

The study highlighted that the most significant predictor of knowledge was the treatment duration, particularly 6-10 years of epilepsy treatment. These findings are consistent with previous studies by Neyaz et al. [17] and Kassie et al. [21], which demonstrated that longer treatment durations were associated with improved knowledge as families became more familiar with symptoms and treatment protocols. In Neyaz et al.'s study conducted in Almadinah Almunawwarah, Saudi Arabia, in 2016, it was found that 44.7% of respondent families believed that epilepsy is related to Jinns (spirits) [17]. However, our study did not delve into this belief, as it appears that by 2024, such notions had declined. In our findings, the age of the respondent, education level, and working status did not significantly affect the knowledge. Elsakka et al. reported a significant association between the level of education and knowledge and attitude (p = 0.038 and p<0.001, respectively) [4]. Shahbo et al. focused on the general population and reported that the level of education in Arar, Saudi Arabia, and Port Said, Egypt, had a positive significant effect on their attitude towards epileptic patients; however, they didn’t assess parental knowledge or attitude [7].

To the best of our knowledge, this is one of the pioneer studies assessing the knowledge and attitudes of parents/family members regarding epilepsy in their children in Arar, Saudi Arabia. The use of PCA significantly enhanced our ability to identify underlying patterns and relationships within the data, while also addressing multicollinearity, thereby improving the robustness and clarity of our findings. Additionally, predictors of knowledge and attitude were identified using multiple linear regression. However, it is important to note that the study has some limitations, including its non-comparative design and relatively small sample size. Further, the adoption of a cross-sectional study design limits our ability to establish causation or observe changes over time in parents' knowledge and attitudes. Also, the use of an online, self-administered questionnaire introduces potential self-selection bias, as it relies on the willingness and ability of participants to respond assuming that respondents have sufficient literacy and internet access further limiting the generalizability of the results.

## Conclusions

Our study highlights a notable disparity between poor knowledge and positive attitudes among the parents surveyed. This underscores the imperative for targeted health education initiatives and awareness campaigns aimed at equipping parents with a comprehensive understanding of their children's condition. Such initiatives should emphasize not only the nature and management of the illness but also its potential long-term impact on their children's quality of life and daily activities. By fostering greater awareness and acceptance, parents can play a pivotal role in supporting their children effectively through their journey with the disease.

## Figures and Tables

**Figure 3:**
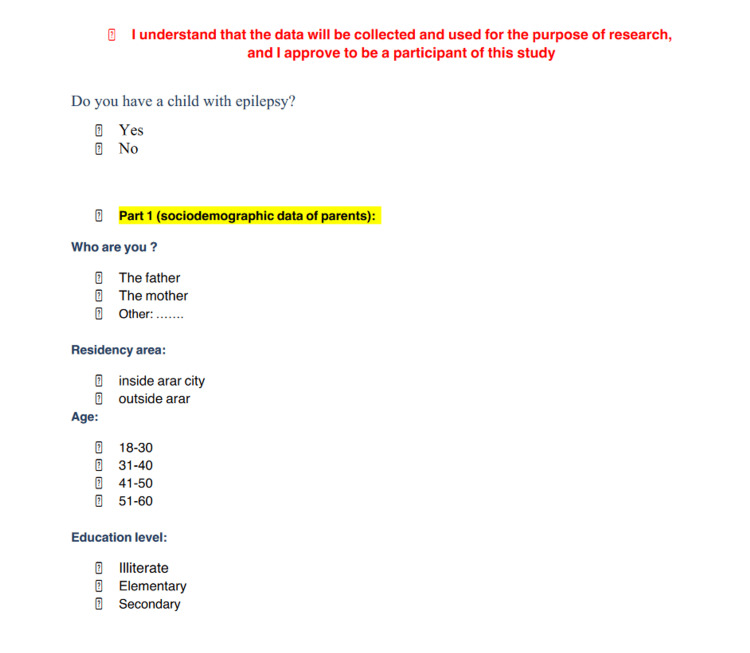
Questionnaire, Page 1

**Figure 4:**
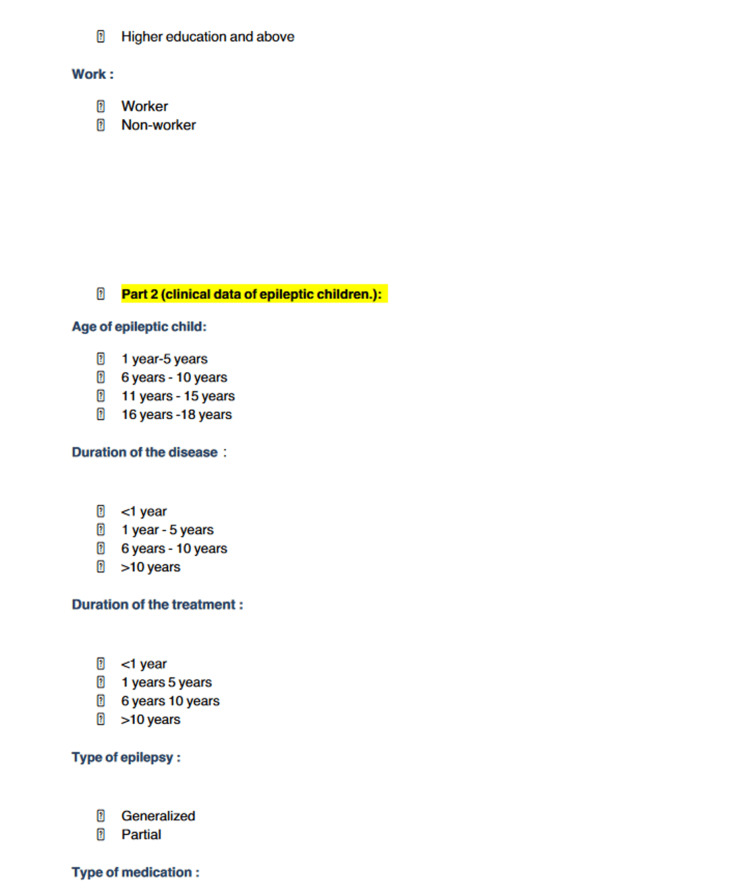
Questionnaire, Page 2

**Figure 5:**
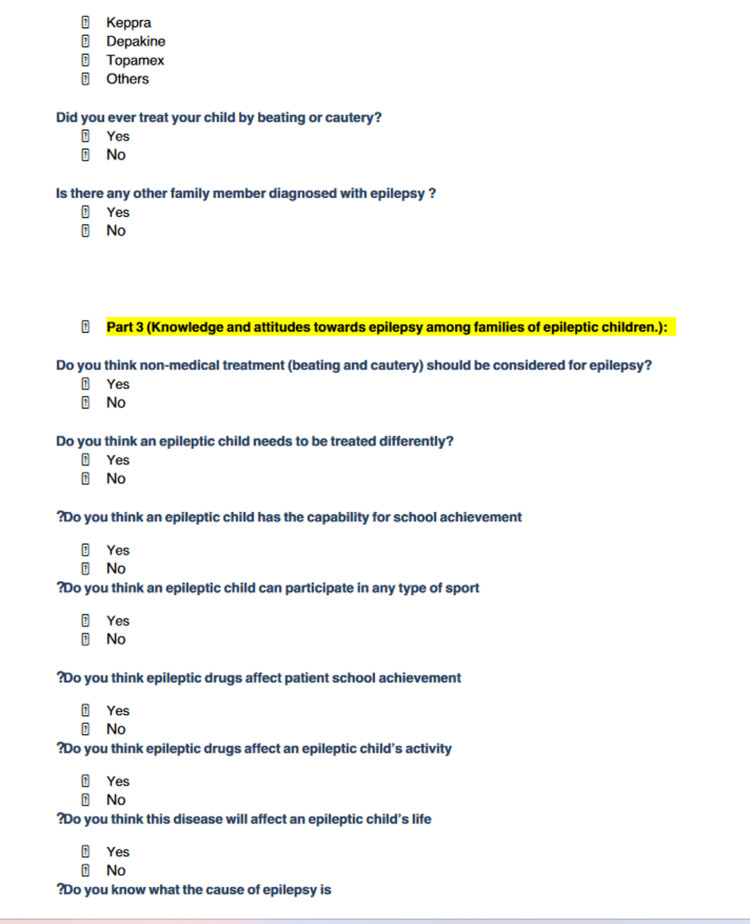
Questionnaire, Page 3

**Figure 6:**
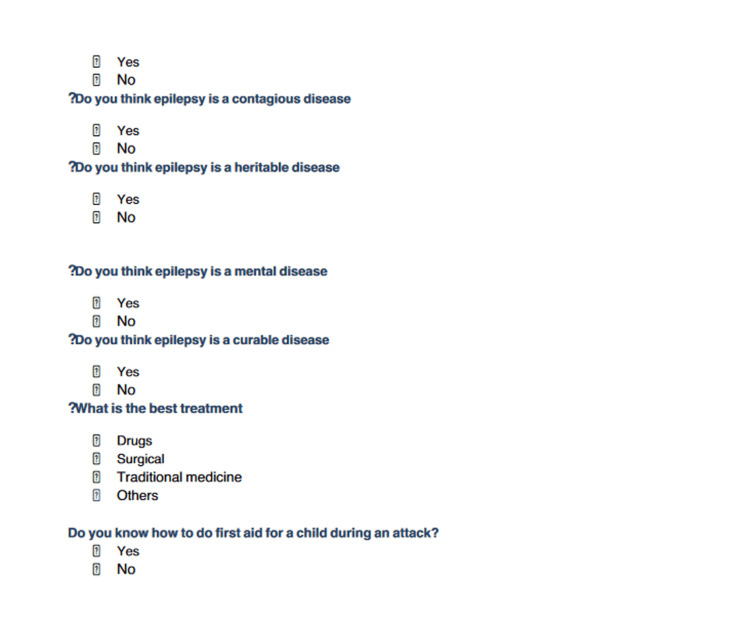
Questionnaire, Page 4

## Appendices

**Table 9:** S1 measures differences if item is deleted from knowledge domain

Item-Total Statistics	Scale Mean if Item Deleted	Scale Variance if Item Deleted	Cronbach's Alpha if Item Deleted
Do you think non-medical treatment (beating and cautery) should be considered for epilepsy?	4.19	2.155	0.580
Do you think that children with epilepsy need special treatment?	3.46	2.142	0.565
Do you think an epileptic child has the capability for school achievement?	3.39	2.406	0.606
Do you think that a child with epilepsy can participate in any type of sport?	3.65	2.176	0.635
Do you think epileptic drugs affect patient school achievement?	3.86	1.625	0.470
Do you think that epilepsy medications affect the child's activity?	3.70	1.609	0.449
Do you think epileptic drugs affect patient life?	3.77	1.631	0.469

**Table 10:** S2 measures differences if item is deleted from attitude domain

Item-Total Statistics	Scale Mean if Item Deleted	Scale Variance if Item Deleted	Cronbach's Alpha if Item Deleted
Do you know what causes epilepsy?	4.66	1.323	0.268
Do you think epilepsy is a contagious disease?	4.97	1.506	0.256
Do you think epilepsy is a hereditary disease?	4.51	1.404	0.327
Do you think epilepsy is a mental illness?	4.72	1.521	0.379
Do you believe that epilepsy is a curable disease?	4.19	1.635	0.346
What is the best treatment?	3.11	1.714	0.453
Do you know what is the correct first aid for a child having an epileptic seizure?	4.24	1.666	0.390
